# Stable microbial community in compacted bentonite after 5 years of exposure to natural granitic groundwater

**DOI:** 10.1128/msphere.00048-23

**Published:** 2023-09-29

**Authors:** Katja Engel, Sian E. Ford, W. Jeffrey Binns, Nikitas Diomidis, Greg F. Slater, Josh D. Neufeld

**Affiliations:** 1 Department of Biology, University of Waterloo, Waterloo, Ontario, Canada; 2 School of Geography & Earth Sciences, McMaster University, Hamilton, Ontario, Canada; 3 Nuclear Waste Management Organization, Toronto, Ontario, Canada; 4 NAGRA, Wettingen, Switzerland; The University of Iowa, Iowa City, Iowa, USA

**Keywords:** MX-80 bentonite clay, nuclear waste disposal, microbial characterization, 16S rRNA gene sequencing, cultivation, PLFA

## Abstract

The Materials Corrosion Test (MaCoTe) at the Underground Research Laboratory in Grimsel, Switzerland, assesses the microbiology and corrosion behavior of engineered barrier components of a deep geological repository (DGR) for long-term disposal of high-level nuclear waste. Diversity and temporal changes of bentonite-associated microbial community profiles were assessed under DGR-like conditions for compacted Wyoming MX-80 bentonite (1.25 g/cm^3^ and 1.50 g/cm^3^ targeted dry densities) exposed to natural groundwater. Using culture-dependent and molecular techniques, samples taken from the outside layer of 5-year borehole modules revealed up to 66% and 23% of 16S rRNA gene sequences affiliated with *Desulfosporosinus* and *Desulfovibrio,* respectively. Putatively involved in sulfate reduction, these taxa were almost undetectable within the bentonite core. Instead, microbial profiles of the inner bentonite core were similar to uncompacted bentonite used to pack modules years earlier, and were consistent with a previously published 1-year time point, revealing no detectable microbial growth. Abundances of culturable aerobic and anaerobic heterotrophic bacteria in the uncompacted bentonite were relatively low, with less than 1,000 and 100 colony-forming units (CFUs) per gram dry weight, respectively. Nearly 5 years after emplacement, culturable heterotrophic bacterial CFUs and sulfate-reducing bacteria did not change significantly inside the bentonite core. Phospholipid fatty acid data indicated similar lipid abundance, and corresponding cell abundance estimates, for inner 5-year MaCoTe bentonite samples compared to those previously obtained for 1-year incubations. Collectively, our results provide complementary evidence for microbial stability inside highly compacted bentonite exposed to conditions that mimic engineered barrier components of a deep geological repository.

**IMPORTANCE:**

The long-term safety of a deep geological repository for used nuclear fuel is dependent on the performance of the engineered and natural barriers. Microbial activity can produce chemical species that can influence the corrosion of the disposal containers for used nuclear fuel. Although previous studies have evaluated the microbiology of compacted bentonite clay within subsurface environments, these have been limited to relatively short incubations (i.e., 1 year). The current study provides a unique 5-year perspective that reinforces previous findings of growth inhibition for bentonite clay exposed to *in situ* subsurface conditions.

## INTRODUCTION

A deep geological repository (DGR) is a multi-barrier system of natural and engineered components designed to permanently store used nuclear fuel and isolate it from the surrounding environment. In the DGR design being considered for Canada, 48 used Canada deuterium uranium fuel bundles will be placed in used fuel containers (UFCs) made of carbon steel with a corrosion-resistant copper coating. The UFCs provide mechanical strength designed to withstand the pressure of swelling clay engineered barriers and a potential hydrostatic head due to future glaciation events (1). Each UFC will be encased in highly compacted bentonite clay buffer boxes in underground emplacement rooms, and void spaces will be filled with processed “gapfill” bentonite. Highly compacted bentonite and gapfill bentonite will swell upon eventual contact with groundwater, sealing the emplacement rooms and generating increased swelling pressures, limited pore space, and relatively low water activity.

Microorganisms within barrier components of a proposed DGR have the potential to influence safety and stability through contributions to metal corrosion, bentonite transformation, gas production, and sorption of radionuclides (2). Sulfate-reducing bacteria (SRB) are of specific interest due to their production of hydrogen sulfide gas (H_2_S), which has potential for UFC metal corrosion. Although previous studies have explored microbial communities present in as-received, uncompacted bentonite clay and lab-scale pressure vessels (3 – 8), few SRB are detected and this may be a result of bentonite-associated inhibition. In particular, highly compacted bentonite can inhibit microbial growth when exceeding 1.6 g/cm^3^ dry density, which corresponds to water activity below 0.96 and swelling pressures exceeding 2 MPa (9), and it was shown that swelling bentonites decrease sulfide production by SRB (10). A study featuring ancient analog samples reported low cultivable heterotroph and SRB associated with a bentonite deposit that was formed ~10 million years ago and sequencing of SRB cultures revealed that the taxon present was affiliated with the genus *Desulfosporosinus* (7). An analysis of dry, uncompacted bentonite clay samples showed that cultivation-based approaches were consistently associated with phylogenetically similar SRB, aerobic heterotrophs, and fermenters (8). Dominant nucleic acid sequences extracted from these dry clay samples did not correspond with the bacteria that were enriched or isolated in culture, and relatively few core taxa (e.g., *Streptomyces*, *Micrococcaceae*, *Bacillus*, and *Desulfosporosinus*) were shared among cultivation and direct nucleic acid analysis profiles (8).

Leveraging samples from the Materials Corrosion Test (MaCoTe) at the Underground Research Laboratory in Grimsel, Switzerland, we explored the diversity and temporal changes of bentonite-associated microbial profiles under DGR-like conditions for compacted Wyoming MX-80 bentonite (targeting 1.25 g/cm^3^ and 1.50 g/cm^3^ dry densities) during exposure to natural groundwater. Similar to a parallel Mont Terri experiment (11, 12), both 16S rRNA gene and phospholipid fatty acid (PLFA) profiles for borehole modules exposed for 13 months were published previously (13). Here, we report microbial community data for bentonite samples collected after 5 years of subsurface emplacement using culture-dependent and molecular techniques.

## RESULTS

### Microbial abundances in bentonite for Year 5 module samples

**Fig 1:**
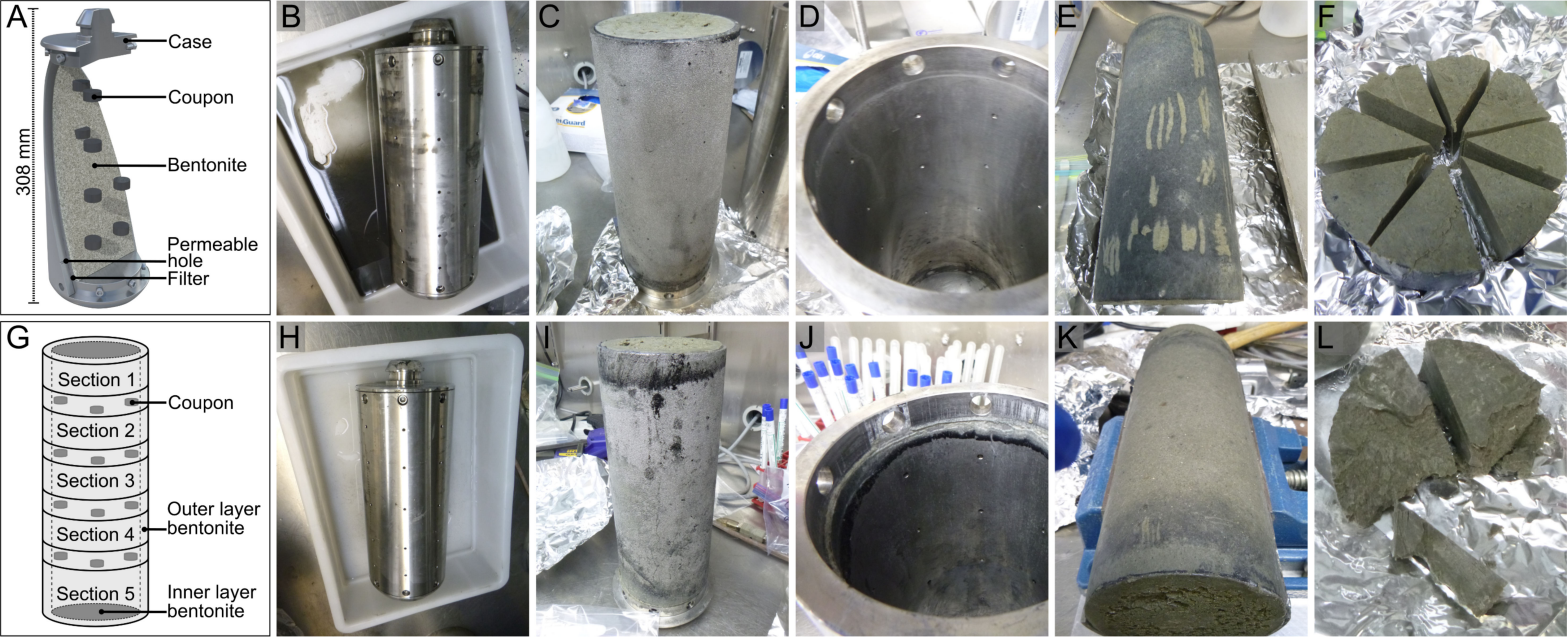
Borehole module design and components. Schematic of the borehole module (**A**) and bentonite core sections (**G**). Pictures of the 1.25 (B–F) and 1.50 (H–L) g/cm^3^ MaCoTe borehole modules during disassembly in an anerobic chamber showing metal case surrounded by transport flask fluid (**B, H**), outside surface of the ceramic filter (**C, I**), inside of the empty metal case (**D, J**), longitudinal view of the bentonite core surface with shallow, ~1 mm-deep sampling marks, and transverse view of bentonite section 5 (**F, L**).

**Fig 2:**
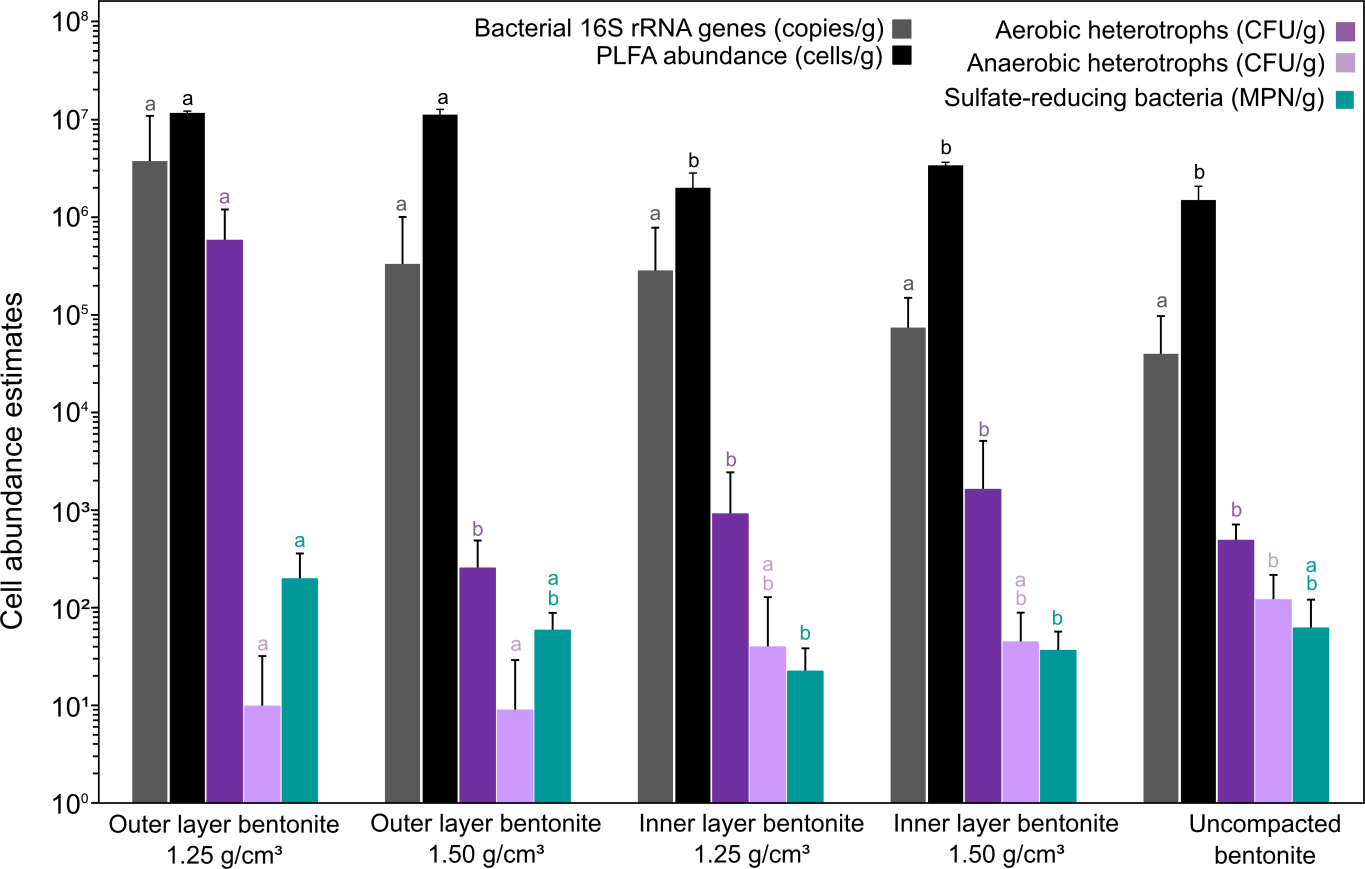
Microbial abundance estimates for outer and inner layers of MaCoTe bentonite samples after 5 years of storage in borehole 13.001 compared to uncompacted bentonite used to prepare the modules. Error bars represent the standard deviation of 5 (culture-based enumerations), 5–8 (qPCR counts), or 2 (PLFA) replicates. Different letters above bars indicate significant differences (Tukey-Kramer test, *p* < 0.05) within each group (i.e., qPCR) only.

A mixture of Wyoming MX-80 bentonites “MX6” and “MX7” was used to pack low- (1.25 g/cm^3^) and high-density (1.50 g/cm^3^) MaCoTe borehole modules for this study (Fig. 1). Both starting materials were recently characterized microbiologically (8). These uncompacted bentonite sources are together considered as “time-zero” starting material, with respect to microbial abundances and identity, and we analyzed a composite of both for the present study. After disassembling borehole modules aseptically (Fig. 1B through F and H through L), cultivable heterotrophic microorganisms were counted on nutrient low Reasoner’s 2A (R2A) agar plates using glucose as energy and carbon source and SRB were enumerated using sulfate-containing media with lactate as electron donor. Microbial abundances were determined for samples obtained from each of five module sections and from both outer and inner layers (Fig. 1G). Culture-dependent abundance estimates showed that, after 5 years emplacement in borehole 13.001, aerobic heterotrophs, anaerobic heterotrophs, and SRBs were at or below abundances detected for the uncompacted bentonite, except for the outer layer of the low-density module (Fig. 2). Although water activity of inner layer bentonite from the low-density module was higher than the high-density module (Table 1), the number of cultivable microorganisms in the inner bentonite did not exceed the high-density module or the starting material. Culture-independent abundance estimates based on PLFA analysis or quantitative PCR (qPCR) were one to three orders of magnitude higher than culture-dependent abundances (Fig. 2). Similar to culture-dependent analyses, the abundance of bacteria determined with qPCR was not significantly higher for inner layer bentonites compared to the starting material. Microbial biomass estimates based on PLFA analysis yielded the highest values for all samples compared to the other tested methods. Although cell abundance estimates for outer layer bentonite material were significantly higher, inner layer bentonite abundances did not differ from uncompacted bentonite (Fig. 2).

**TABLE 1:** Water activity and moisture content of uncompacted bentonite (composite of MX6 and MX7) which was used to pack Modules 3 and 4 before their emplacement in borehole 13.001 for 5 years

Sample^*a*^	Clay type	Targeted dry density (g/cm^3^)	Moisture (%)	Water activity
Uncompacted^#^	MX-80 bentonite	N/A	5.96 ± 0.04	0.415 ± 0.003
Module 3*	MX-80 bentonite	1.25	30.75 ± 1.30	0.983 ± 0.006
Module 4*	MX-80 bentonite	1.50	24.04 ± 1.03	0.954 ± 0.005

^*a*^ Standard deviation of six (^#^) or five (*) replicates is shown.

### Microbial diversity in bentonite for Year 5 module samples

By using negative control data for comparison, the decontamination step identified a total of 59 amplicon sequence variants (ASVs; of 3,562 ASVs total) as probable contaminants (Fig. S1), with one common ASV affiliated with *Staphylococcus* (ASV#749) identified in each of three batches of DNA extractions but more predominantly in Year 5 samples and controls. Furthermore, five ASVs (*Acinetobacter lwoffii* ASV#1077, *Pinus koraiensis* ASV#1272, *Comamonadaceae* ASV#1286, *Cutibacterium* ASV#1999, Bacteria ASV#2045) were identified in two of the DNA isolation kit control extraction data but with relatively low abundances in both Year 1 and Year 5 samples. All contaminant ASVs were removed from the data before subsequent analyses.

**Fig 3:**
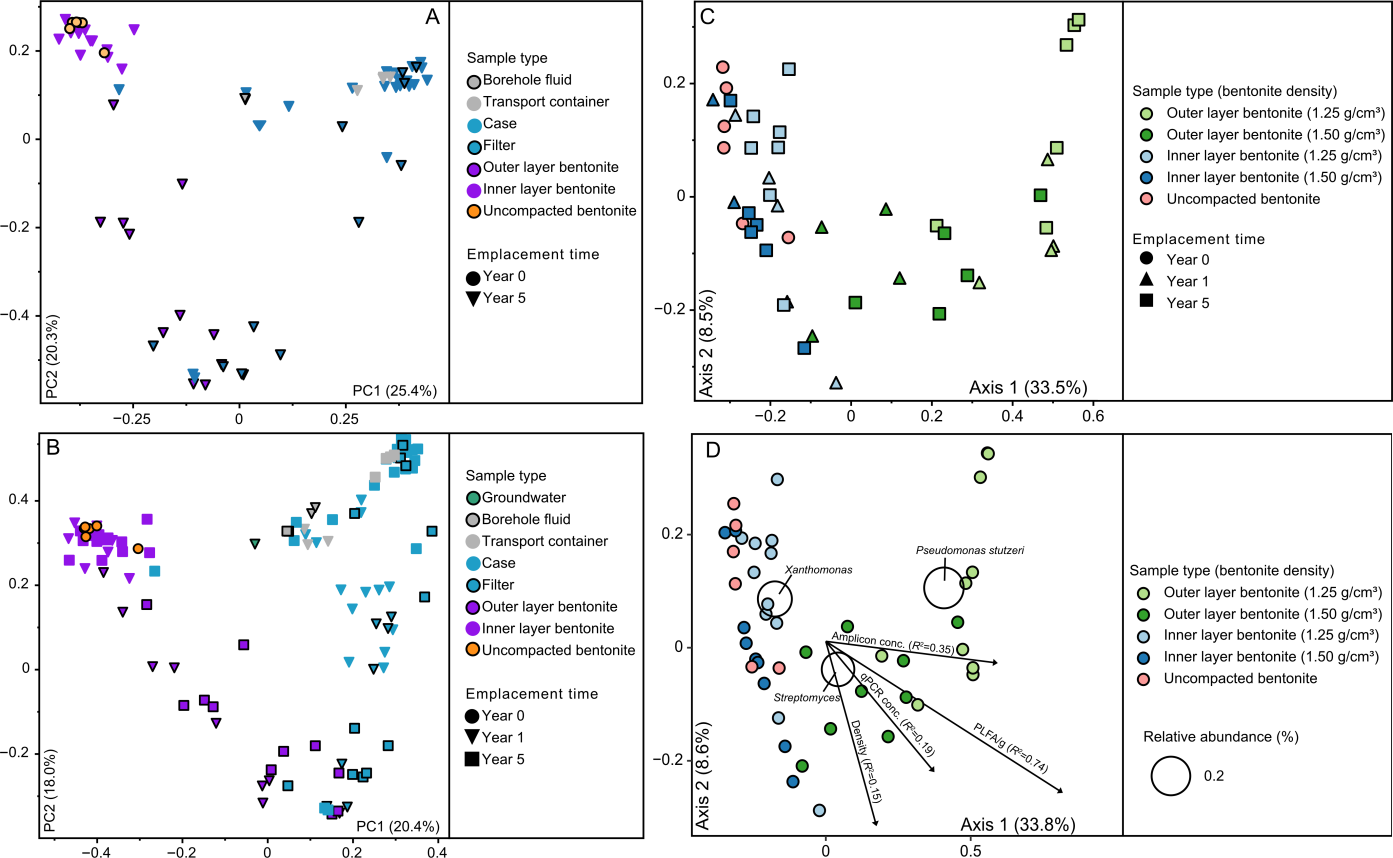
Grouping of borehole module samples and uncompacted bentonite in a principle coordinate analalysis (PCoA) ordination based on Bray-Curtis metric. Plots were generated including samples emplaced in borehole 13.001 for 5 years (**A**) or 1 and 5 years (**B, C, D**). Bentonite and borehole module-associated samples were shown (**A, B**) or bentonite only (**C, D**). Triplot (**D**) shows species at or above 0.1% relative abundance (open circles) located based on relative abundance-weighted average of PCoA coordinates of samples in which the taxa were present. Only metadata variables with *R* ^2^ values above 0.1 were shown. Samples were rarefied to 2,000 reads. Uncompacted bentonite (Year 0) used to prepare the borehole modules is shown in all plots.

**Fig 4:**
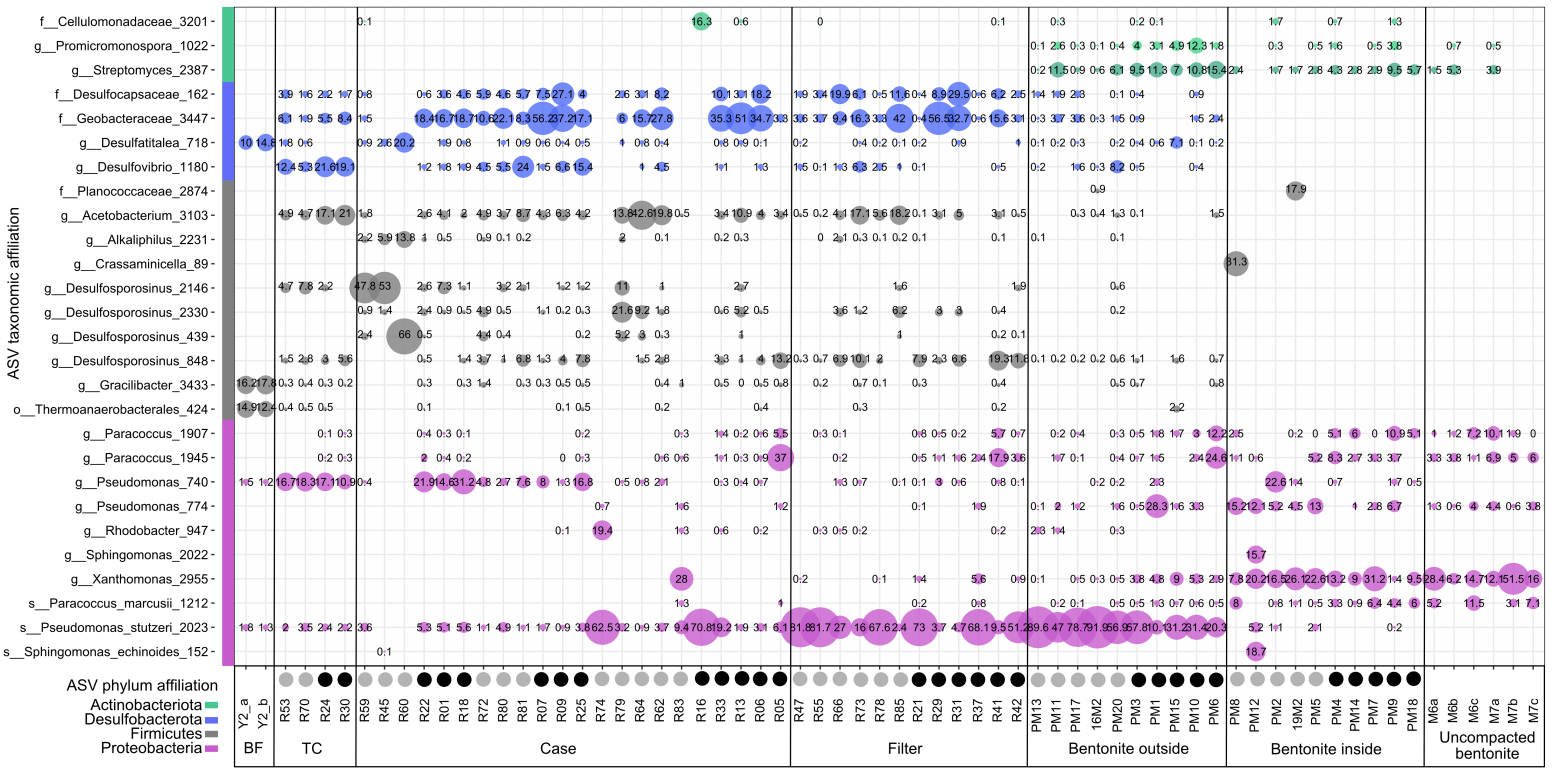
Bubble plot showing 16S rRNA gene profiles of borehole module and bentonite samples after 5 years of exposure in borehole 13.001. Only ASVs at or above 10% abundance are shown. Samples are sorted from the outside [BF, transport container (TC)] to the inside of the 1.25 g/cm^2^ (gray circles) and 1.50 g/cm^2^ (black circles) dry density borehole modules. The 16S rRNA gene profiles of uncompacted bentonite used to prepare the borehole modules are shown on the far right.

**Fig 5:**
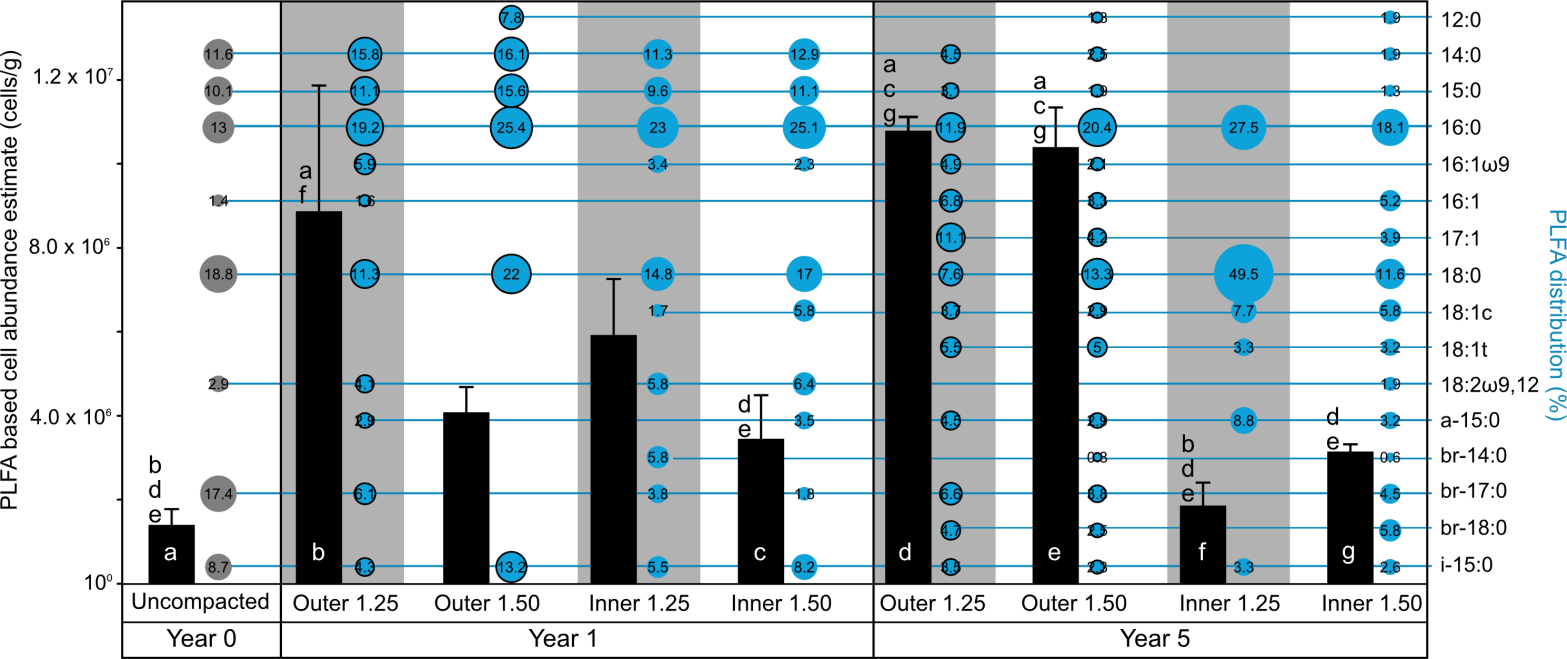
PLFA based cell abundance estimates and PLFA profiles in bentonite samples based on inner and outer layer bentonite after 1 or 5 years of emplacement in borehole 13.001, compared to uncompacted bentonite that was used to prepare the borehole modules. Only PLFAs at or above 5% abundance are shown. Error bars represent the standard deviation of two replicates. Letters above bars indicate significant differences to the sample(s) with the same white letter within the bar (Tukey test, *p* < 0.05).

Samples from borehole modules containing bentonite at dry densities of 1.25 g/cm^3^ and 1.50 g/cm^3^ were collected to analyze microbial community composition using 16S rRNA gene sequencing, including swabbed outer layers in contact with borehole fluid (BF) (e.g., case, filter), outer bentonite core, and inner bentonite core (Fig. 1A and G). Based on amplicon abundance profiles, an ordination analysis showed that uncompacted and inner layer bentonite core samples grouped together and were separated from borehole fluid and case samples (Fig. 3A). Although borehole fluid samples were dominated by ASVs affiliated with *Desulfatitalea*, *Gracilibacter*, and *Thermoanaerobacterales,* samples from case and filter swabs were dominated by ASVs affiliated with *Desulfocapsaceae, Geobacteraceae, Acetobacterium, Desulfosporosinus,* and *Pseudomonas stutzeri* (Fig. 4). Outer layer bentonite sample profiles were dominated by *Pseudomonas stutzeri*, representing up to 92% ASV relative abundance. In contrast, uncompacted bentonite material was dominated by ASVs affiliated with *Xanthomonas* and this same ASV was most abundant in inner layer bentonite samples (Fig. 4). No significant differences were detected between bentonite microbial profiles of inner layer and uncompacted bentonite samples (Fig. S2H). Similarly, no differences were detected between inner or outer layer samples of low- and high-density borehole modules (Fig. S2E and F). However, ASVs affiliated with *Pseudomonas stutzeri* (ASV #2023) and *Streptomyces* (ASV #453) were significantly more abundant in outer layer bentonite sample profiles for both low- and high-density module samples compared to uncompacted bentonite profiles (Fig. S2G). PLFA distributions were generally similar for Year 1 and 5 inner layer bentonite samples, though the high-density Year 5 sample also had a higher diversity of PLFA similar to that observed for the Year 5 outer samples (Fig. 5). C18:2w9,12 which has been associated with plants and fungi (14) was detected at low abundances in some samples, including the Year 5 high-density inner sample. However, 18S rRNA gene PCR did not yield an amplicon for bentonite samples (Fig. S3).

### Identity of cultivable microorganisms in year 5 borehole module samples

**Fig 6:**
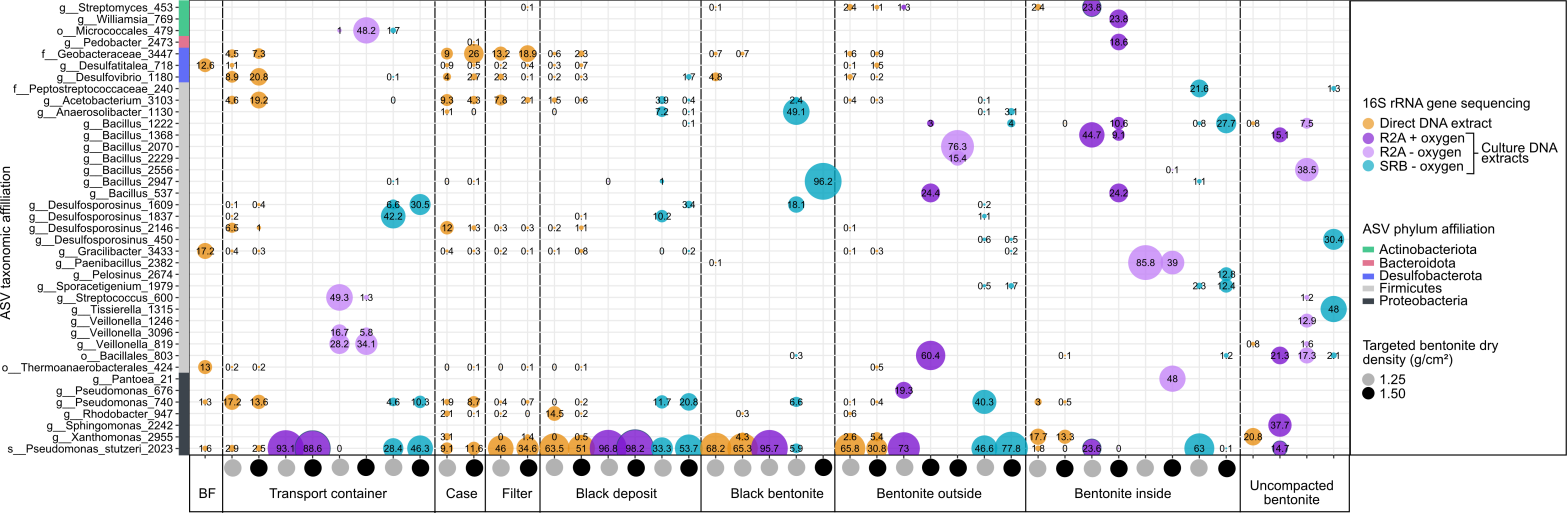
Bubble plot showing 16S rRNA gene profiles of borehole module samples after 5 years of exposure in borehole 13.001 and corresponding cultures, if applicable. Only ASVs at or above 10% abundance are shown. Samples are sorted from the outside (BF) to the inside of the 1.25 g/cm^3^ (gray circles) and 1.50 g/cm^3^ (black circles) dry density borehole modules. The 16S rRNA gene profiles of replicate samples of direct DNA extracts (orange circles) and cultures for aerobic (dark purple circles), anaerobic (light purple circles) heterotrophic and sulfate-reducing (turquoise circles) microorganisms were merged.

Following the culturing of transport flask fluid (Fig. 1B and H), black deposit (Fig. 1J), black bentonite (Fig. 1E and K), inner and outer layer bentonite (Fig. 1G), and uncompacted bentonite in lactate/sulfate-containing media or on low nutrient R2A agar plates, DNA was extracted from liquid culture or colonies and analyzed by 16S rRNA gene sequencing to identify cultivated microorganisms. Although distinct ASVs were associated with cultures from different borehole module locations, low- and high-density borehole module samples often revealed similar cultivated microorganisms for a given compartment (Fig. 6). The ASVs affiliated with *Pseudomonas* and *Bacilli* were commonly found in DNA extracts from cultures and these taxa were not the dominant ASVs detected in direct DNA extracts from the same sample materials. *Xanthomonas,* a Gram-negative, aerobic organism, which is often associated as plant pathogen (15), was most abundant in uncompacted bentonite using 16S rRNA gene sequencing of direct DNA extracts (21%, Fig. 6) but no reads associated with this ASV were identified in aerobic R2A cultures. Incubation of uncompacted bentonite on oxic R2A plates resulted in detection of nine ASVs by 16S rRNA gene sequencing and the most abundant ASVs were associated with *Sphingomonas*, *Bacillales* (ASV#803 and 1368), and *Pseudomonas stutzeri* (Fig. 6), which were not detected in profiles from direct sample DNA extractions (Table S1). Even though detected only by cultivation, ASV#803 was associated with profiles for all three cultivation conditions (i.e., aerobic heterotroph plates, anaerobic heterotroph plates, and SRB tubes). From 16 ASVs detected in uncompacted bentonite cultured on R2A under anoxic conditions, ASVs affiliated with *Veillonella* and *Bacillus* were also present in profiles from direct sample DNA extractions. For SRB media cultivation, none of the 20 detected ASVs were present in the direct DNA sequencing sample profiles. The most abundant ASVs detected in SRB cultures of uncompacted bentonite were *Tissierella* (ASV#1315) and *Desulfosporosinus* (ASV#450) (Fig. 6). *Pseudomonas* (ASV#2023), which was abundant in direct DNA sequencing of case, filter, and outer layer bentonite samples, was also cultured on R2A plates and SRB media (Fig. 6).

### Microbial community profile comparison for Year 1 and Year 5 samples

**Fig 7:**
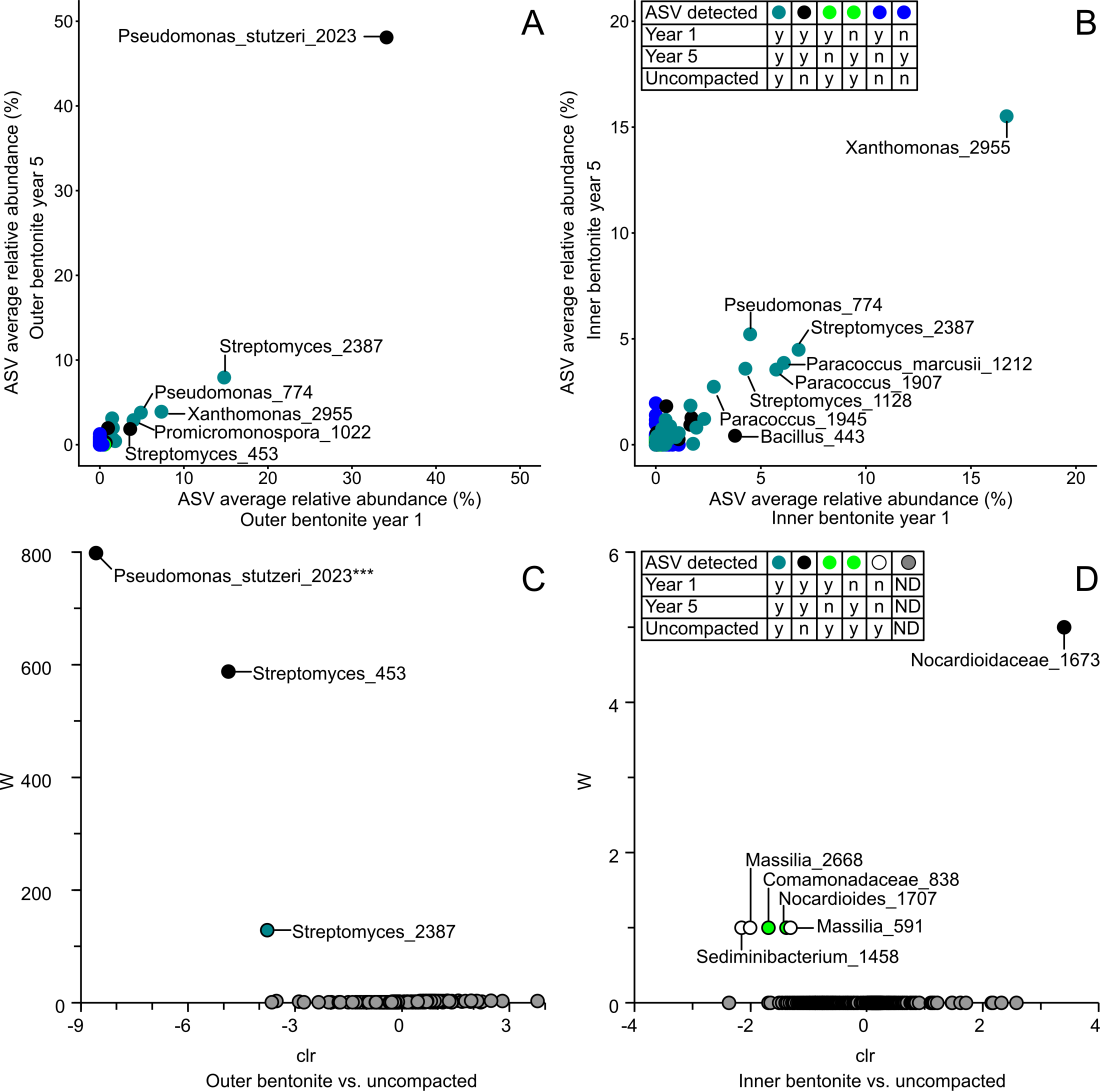
Differential abundances in outer, inner, and uncompacted bentonite samples. Scatter plot of ASVs detected in inner (**A**) or outer (**B**) bentonite samples after 1 and 5 years of exposure. Analysis of composition of microbiomes differential abundance analysis of uncompacted bentonite versus Year 1 and Year 5 inner (**C**) or outer (**D**) bentonite. ASV #2023 (**D**) showed a statistically significant difference in abundance (*p* < 0.05, ***) between uncompacted and outer layer bentonite from Years 1 and 5. ASVs which were present in the borehole modules and uncompacted bentonite are highlighted in dark green and black if absent.

The 16S rRNA gene data for borehole 13.001 modules after 1 year emplacement were reported previously (13), based on amplification and sequencing with prokaryotic primers (16). To compare Year 1 and Year 5 samples, and assess the robustness of earlier results to primer changes, DNA extracts from Year 1 samples were amplified and sequenced alongside Year 5 samples using a universal V4–V5 primer pair. The comparison revealed highly similar ordination space groupings for profiles generated by both primer pairs (Fig. S4). Taxa affiliated with *Xanthomonas*, *Streptomyces,* and *Paracoccus* dominated uncompacted bentonite profiles generated with both primer pairs [Fig. S6; (13)]. Both Year 1 and Year 5 samples from equivalent borehole module locations grouped within ordination space, regardless of emplacement duration (Fig. 3B). For both time points, inner layer bentonite samples grouped with uncompacted starting material samples (Fig. 3B and C) and ASVs affiliated with *Xanthomonas* were relatively abundant in all profiles (Fig. 3D). The outer and inner layer bentonite appeared similar for Year 1 and Year 5 samples. Several ASVs affiliated with *Pseudomonas stutzeri* and *Streptomyces* dominated outer layer bentonite samples for both time points (Fig. 7A) and no significant differences were observed (Fig. S2I; Table 2). Taxa affiliated with *Xanthomonas* were the most abundant ASV associated with inner bentonite samples for both time points (Fig. 7B) and no significant differences were observed for these samples for Year 1 and Year 5 samples (Fig. S2J). Furthermore, both inner layer sample profiles did not differ from uncompacted bentonite (Fig. S2D; Fig. 6H and L).

**TABLE 2:** ANCOM analysis^*a*^

Sample type	Year	ASV taxonomy (ASV #)	*p* value
Outer 1.25 vs 1.50	1	*Streptomyces* (#1128)	<0.05
Inner 1.25 vs 1.50	1	–	–
Outer vs uncompacted	1	*Pseudomonas stutzeri* (#2023)	<0.05
Inner vs uncompacted	1	–	–
Outer 1.25 vs 1.50	5	–	–
Inner 1.25 vs 1.50	5	–	–
Outer vs uncompacted	5	*Pseudomonas stutzeri* (#2023) *Streptomyces* (#453)	<0.05
Inner vs uncompacted	5	–	–

^*a*^ ANCOM, analysis of composition of microbiomes.

After removal of Year 5 borehole modules, fluid from borehole 13.001 was dominated by taxa affiliated with *Firmicutes* and *Desulfobacterota* (Fig. S5). The most abundant ASVs assigned to the *Firmicutes* were associated with *Thermoanaerobacterales* (~14%) and *Gracilibacter* (~25%). The dominant *Desulfobacterota* ASV was associated with *Desulfatitalea* (~12%). For Year 1 profiles, predominant ASVs were associated with *Desulfosporosinus* (ASV#6) and *Desulfovibrio* (ASV#1180), which were not detected in Year 5 borehole fluid samples (Fig. S5); ASVs associated with *Smithella* (ASV#85) were detected at low relative abundance.

The PLFA profiles for Year 1 bentonite samples were re-analyzed alongside uncompacted bentonite and Year 5 samples to facilitate the time point comparison. No significant differences in PLFA-based cell abundance estimates were detected between uncompacted and inner bentonite samples after 1 and 5 years of emplacement. Furthermore, similar PLFA profiles were detected in samples of uncompacted and Year 1 inner and outer bentonite layer samples (Fig. 5). Although PLFA profiles differed between uncompacted and Year 5 inner bentonite samples, no significant changes in PLFA-based cell abundances were detected. Cell abundance was significantly higher for outer layer bentonite samples from Year 5 and 1.25 g/cm^3^ Year 1 samples compared to uncompacted bentonite (Fig. 5).

## DISCUSSION

Microbial persistence within highly compacted bentonite is an important design consideration for DGRs given the potential for microbiologically influenced corrosion of UFC metals. Regardless of bentonite dry density, our cultivation-independent biomass abundance estimates reveal that inner bentonite samples, with most relevance to a DGR given proximity to UFCs, do not differ from uncompacted starting material after 5 years of subsurface exposure (Fig. 2). In addition, our cultivation-dependent abundance estimates of heterotrophs and SRB show no significant differences between uncompacted and inner bentonite samples for both modules (Fig. 2). Demonstrating similar starting material to those analyzed previously, our PLFA, qPCR, and cultivable microorganism abundance estimates for MaCoTe uncompacted bentonite were similar to other MX-80 bentonites analyzed previously (8, 9). Although increasing bentonite density decreased the abundance of cultivable microorganisms in previous controlled lab incubations (3, 9, 10, 17), our data reveal that 1.25 g/cm^3^ dry density modules had similar microbial abundances to the 1.50 g/cm^3^ modules. Borehole modules comparable to our design at the Mont Terri site, exposed to natural groundwater for 1.5 and 5.5 years, showed lower microbial abundances for high-density modules at the earlier time point (11). However, after 5.5 years of exposure, abundances of aerobic heterotrophs, anaerobic heterotrophs, and SRB were not statistically different (11). The PLFA-based abundance estimates for Year 1 inner bentonite samples also indicated that there were no significant differences between low- and high-density modules (Fig. 5). Abundance estimates varied based on method, with PLFA-based values always being higher than qPCR (Fig. 2), which might be attributed to the qPCR primers targeting only bacterial 16S rRNA genes. Culture-dependent enumerations were lower than qPCR, which can be attributed to detection of multiple 16S rRNA gene copies per genome, or relic DNA in qPCR, or by cultivation selecting for a subset of viable taxa (8). One discrepancy in our data is an approximate threefold increase of aerobic heterotrophs for the 1.25 g/cm^3^ outer layer sample (Fig. 2), likely attributed to *Pseudomonas* growth (Fig. 4), that was not reflected by increased PLFA or qPCR values for this sample (Fig. 2). Future research should focus on distinguishing among biomarker detection limits given the background presence of relic material from non-viable cells or cell debris.

Several ASVs affiliated with *Xanthomonas, Streptomyces, Pseudomonas*, and *Paracoccus* were most abundant in inner bentonite samples from Year 1 and Year 5 samples (Fig. 4), which have been reported previously for MX-80 bentonite (8, 11, 18 – 21). Although our previous results showed that ASVs associated with *Streptomyces* and *Xanthomonas* dominated uncompacted and inner bentonite samples analyzed for Year 1 samples (13), our current data set shows *Xanthomonas* ASVs as being more abundant and prevalent than *Streptomyces* ASVs (Fig. S6). This difference could be attributed to amplification bias given that a nested PCR approach with 50 cycles of amplification was used previously (13), whereas the current study used a non-nested approach with only 45 cycles of amplification. Because DNA yield and microbial community profiles can be strongly influenced by the choice of DNA extraction method and/or PCR primers (22, 23), this reinforces the importance of methodological consistency across samples to allow for direct comparisons. Nonetheless, despite relatively minor differences in relative abundances among dominant taxa, overall conclusions related to microbial community composition and sample beta diversity were robust to distinct biases associated with primer set and PCR amplification conditions.

Although Year 1 and 5 borehole fluid showed different microbial profiles, with only few overlapping ASVs, taxa affiliated with *Firmicutes* and *Desulfobacterota* dominated for both time points (Fig. S5). ASV#3433 was associated with *Gracilibacter* (Fig. S5) and showed 98% nucleotide identity to *Gracilibacter thermotolerans*, which is an anaerobic and thermotolerant bacterium (24). However, the strain was shown not to grow at or below 20°C, which would be above the temperature of the borehole (14°C–16°C, Table 3). ASV#718 associated with *Desulfatitalea* showed 98% identity to *Desulfatitalea tepidiphila*, an SRB isolated from tidal flat sediment, which was shown to grow at 13°C to 42°C, using acetate and lactate and other substrates, as well as autotrophic growth with H_2_ (25). No cultured representative with >98% identity was identified for ASV#424 associated with *Thermoanaerobacterales*. Abundant microorganisms detected in the borehole fluid were absent from inner bentonite samples, indicating that swelling pressures generated by 1.25 g/cm^3^ and 1.50 g/cm^3^ dry weight bentonite succeeded in preventing microbial movement to the inside or their survival. Similar to our findings, previous research (11) detected a change in the borehole microbial composition after 1.5 and 4.5 years of emplacement (11), potentially due to disturbance of the borehole associated with the deployment of experiments. The authors argued that the borehole microbial community reached a steady state after 5.5 years of employment (11); however, the addition of new borehole modules throughout the experiment in our borehole 13.001 could potentially further promote changes in the microbial community composition.

**TABLE 3:** Borehole 13.001 water chemistry^*a*^

Measurement	T (°C)	pH	EC (mS/cm)	Eh (mV)	O_2_ (ppm)
23 April 2014	14.2	8.4	0.1	−60	<0.7
9 November 2021	16	8.32	3.93	−140 to −155	Not determined
30 November 2022	16.1	8.17	4.09	−140	Not determined

^*a*^ Temperature (T), pH, electrical conductivity (EC), redox potential (Eh), and oxygen (O_2_) concentration at different time points. Data provided by Nagra.

Cultivation-dependent and cultivation-independent microbial profiles were generated for borehole module components. Most dominant taxa associated with 16S rRNA gene profiles of direct DNA extracts were often undetected using the three cultivation-based conditions (Fig. 6). The absence of ASVs detected in direct DNA extracts from cultivation efforts for bentonite and subsurface rock samples has been reported previously (7, 8). Such discrepancies are expected and may be due to DNA extraction bias (e.g., DNA absorption to clay, incomplete lysis, relic DNA), amplification (e.g., primers, amplification conditions), sequencing (e.g., insufficient coverage), but most importantly culture conditions (e.g., unknown nutritional requirements). For example, the enumeration of SRB was performed using media with lactate as electron donor, but autotrophic SRB capable of using hydrogen gas as electron donor could have been missed. The importance of hydrogen gas within repository-relevant conditions (26, 27) suggests the inclusion of such culture conditions in future sampling campaigns. However, sequencing of 16S rRNA genes from clay samples and cultures provides confidence that most detected SRB were cultivated in most probable number (MPN) tubes. Furthermore, fast-growing organisms like *Pseudomonas* (*Proteobacteria*) and *Bacillus* (*Firmicutes*) can outcompete other phyla in enrichment cultures and incubations mimicking the groundwater environment are planned for future analyses.

Due to culture limitations, differences between cultivation-dependent and cultivation-independent profiles are inevitable but the subset of cultivated microorganisms can highlight potentially active and viable microorganisms that may be relevant for a DGR once bentonite barriers become saturated. In particular, an ASV associated with *Pseudomonas stutzeri* was cultivated using oxic R2A and anoxic SRB incubations, comprising 6%–98% of sequences in transport container, black deposit, black bentonite, and outer bentonite samples for low- and high-density modules (Fig. 6). This same ASV was also detected for inner bentonite samples of the low-density (1.25 g/cm^3^) module using oxic R2A and anoxic SRB cultivation conditions but was undetected (or below 0.1% relative abundance) in the high-density (1.50 g/cm^3^) module (Fig. 6). *Pseudomonas stutzeri* is a Gram-negative facultative anaerobic bacterium, possibly able to use nitrate as electron acceptor under anoxic conditions (28). The presence of ASVs associated with *Pseudomonas stutzeri* in DNA extracts from anoxic cultivations could be a result of trace amounts of nitrate present in the peptone or meat extract (29) used in the R2A or SRB media. Alternatively, it could indicate that small amounts of oxygen were present in the cultures, possibly bioavailable in association with clay (11) that was included within culture tube inoculations, using that oxygen to metabolize available organic carbon before anoxia was established. However, indicator strips used to monitor the incubations suggested that anoxic conditions were established within the head space and the detection of ASVs associated with the strictly anaerobic genera *Desulfosporosinus* (30) in SRB media cultures implies very little oxygen was available. Even though *P. stutzeri* was predominantly identified at the outside of highly compacted bentonite cores, these bacteria could nonetheless contribute to dinitrogen gas through denitrification, potentially leading to fissures within the DGR if sufficient partial pressure is produced. Previous research detected *Pseudomonas* spp. within bentonite enrichment cultures (17, 19, 20, 31) but are unlikely to migrate within >1.68 g/cm^3^ dry density bentonite (32, 33). Despite being non-spore forming, *P. stutzeri* appears to survive high pressure and temperature conditions (20, 21), possibly by being desiccated and inactive. Burzan and colleagues (11) observed darker bentonite and black spots, presumably due to ferrous sulfide, and subsequent 16S rRNA gene sequencing showed a dominance of taxa affiliated with *Pseudomonadaceae*, *Desulfobulbaceae*, and *Pseudonocardiacaea*. Our 16S rRNA gene sequencing and culturing results showed that black deposit and black bentonite were dominated by *Pseudomonas*-affiliated ASVs (Fig. 6). Microorganisms cultivated from Year 5 high-density inner bentonite samples include putative spore former *Firmicutes*, such as *Bacillus, Sporacetigenium, Paenibacillus*, and *Pelosinus,* which have been previously identified in iron-reducing MX-80 bentonite enrichments (34). Bacteria affiliated with *Pedobacter* were previously identified in anoxic incubations of Czech bentonite-concrete suspensions (35). An ASV associated with *Pantoea* is closely related to *Pantoea agglomerans*, a reported plant pathogen that was previously identified in cultures from Spanish clay (36).

Most ASVs detected on the outside of the bentonite or on the borehole module were absent from the 1.25 g/cm^3^ and 1.50 g/cm^3^ bentonite cores, likely due to high swelling pressure, small pore sizes, and low water activity, preventing microbial movement and activity in close vicinity to the embedded metal coupons, which is important for long-term safety assessment of the DGR concept. However, microbial activity on the outside of the bentonite core in regions of low or no bentonite pressure can potentially affect the DGR by production of gas, diffusion of corrosive metabolites, or dissolution of the clay (37). We also observed that ASV#3447, associated with *Geobacteraceae* that are known iron-reducing microorganisms, was relatively abundant in case and filter samples. Mineralogical changes of the clay and possible decrease in swelling could affect the sealing properties of the bentonite (38, 39). In addition, ASV#162 was associated with *Desulfocapsaceae,* a family containing five genera of strict anaerobic bacteria capable of various metabolisms including sulfate reduction or disproportionation (40, 41). No cultured representative with >98% identity was identified for this ASV, which limits predictions of putative function, and no growth in SRB medium was observed for our samples. Several ASVs associated with *Desulfosporosinus* (strict anaerobes, sulfate-reducing, spore forming) were detected in the transport container fluid, case, filter, and outer bentonite samples. *Desulfosporosinus* were detected in MX-80 bentonite previously (19) and enrichment up to 30% in uncompacted bentonite demonstrates that these cells are viable and can become active when no swelling pressure is present. To assess the potential role of detected ASVs within a DGR environment, a clear understanding of their metabolic potential is needed. Characterization of isolates and activity studies are important next steps for future studies.

### Conclusions

Long-term safety of a deep geological repository for used nuclear fuel is dependent on the integrity of both engineered and natural barriers, and highly compacted MX-80 bentonite is an important construction material planned for the engineered barrier system. The long-term *in situ* experiment conducted at the Grimsel underground laboratory tested borehole modules with 1.25 g/cm^3^ and 1.50 g/cm^3^ targeted dry densities, monitoring microbial changes in borehole fluid and borehole module components under conditions that mimic a deep geological repository. Our results demonstrate that inner bentonite samples did not change over the 5-year period and the number of cultivable microorganisms in the inner layer of low (1.25 g/cm^3^) and high (1.50 g/cm^3^) dry density modules do not exceed background levels seen for uncompacted bentonite, providing evidence for microbial stability inside highly compacted bentonite due to low water activity, small pore size, and high swelling pressure. *Pseudomonas stutzeri* was abundant on the outside of the compacted bentonite core, indicating that regions of a DGR with relatively low swelling pressure, such as interfaces, may permit some microbial proliferation. Consequently, any DGR design should minimize interfaces with potential for low-density bentonite effects and account for any interface-associated microbial activity when calculating corrosion allowances.

## MATERIALS AND METHODS

### Borehole module sampling

Eight stainless steel borehole modules were assembled as described previously (13, 42) and emplaced in a 9 m-deep vertical borehole (13.001) at the Grimsel Underground Research Laboratory on 22 September 2014. Two modules were removed after 13 months of exposure and Year 1 results were reported previously (13, 42). On 10 April 2019, after ~5 years of exposure (4 years, 6 months, 19 days), Modules 3 (1.25 g/cm^3^) and 4 (1.50 g/cm^3^) were removed from the borehole and placed in transfer flasks along with a borehole fluid while maintaining anoxic conditions following procedures described elsewhere (42). The transfer flasks were transported to Wood PLC laboratories (Harwell, UK) for disassembly. Borehole 13.001 fluid (1.2 L) was filtered on the day of module removal using a 0.22 µm Sterivex filter (MilliporeSigma), which was stored frozen until DNA extraction. Borehole modules were opened and sampled in an anoxic glove box as previously described (13, 42). In the absence of flame, sampling knives were treated with 4.6% bleach to remove DNA, followed by a 96% ethanol rinse. Bentonite cores were cross-sectioned, resulting in five bentonite sections, excluding layers with coupons (Fig. 1G). Bentonite sections were sealed in two Mylar bags before exiting the chamber and stored at −20°C for PLFA and DNA analysis, or at 4°C for cultivation-dependent analysis. Fluid present in each transport flask was filtered (150 mL) using a 0.22 µm Sterivex filter and stored at −20°C until DNA extraction. Swabs (~10 cm^2^ area; Puritan, ME, USA) were taken from several locations within the borehole module for microbiological analysis and stored at −20°C until processing. Uncompacted Wyoming MX-80 bentonite, which was used to prepare the borehole modules in 2014 (MX6 and MX7), was stored at room temperature and a composite of both materials was analyzed along compacted bentonite samples from Modules 3 and 4. Wood PLC performed corrosion analysis of metal coupons and results will be published elsewhere. Here, we report on the identity and abundance of microorganisms using culturing, PLFA, and DNA-based analyses of bentonite and other environmental samples related to MaCoTe borehole Modules 3 and 4.

### Water activity and moisture content

Water activity of bentonite samples was determined using the fast mode on the WP4 Potentiometer (Meter Group, USA) following the manufacturer’s instructions. The instrument was calibrated before each use with a solution of known water potential (0.5 M KCl). Water activity was calculated using the following equation:


$$water\;activity\;=exp\frac{water\;potential*\;molecular\;weight\;of\;water}{temperature*\;gas\;constant}$$

Subsequently, bentonite samples were heated at 110°C for 24 h to determine moisture content using the following equation:


$$moisture\;content\;\left(\%\right)=\left(\frac{wet\;weight-dry\;weight}{wet\;weight}\right)\times 100$$

### Enumeration of cultivable microorganisms in bentonite

Culturable heterotrophic bacteria and SRB in bentonite were enumerated as previously described (8, 13). Bentonite samples (2 g) were suspended in phosphate-buffered saline (18 mL) and mixed in a rotating incubator (15 rpm, 30 min) at room temperature. Suspensions were 10-fold serial diluted in phosphate-buffered saline. Cultivable aerobic and anaerobic heterotrophic bacteria were enumerated on triplicate, low nutrient R2A agar plates (M1687; HiMedia Laboratories) which contain glucose as energy and carbon source. Agar plates were incubated in the presence (7 days) or absence (28 days) of oxygen at 30°C. Cultivable SRB were incubated under anoxic conditions (28 days) at 30°C and enumerated using a five-tube MPN method, using lactate and sulfate-containing SRB medium (M803; HiMedia Laboratories). For each enumeration of cultivable microorganisms, the average of five biological replicates was determined.

### PLFA extraction and analysis

Lipids were extracted twice from lyophilized bentonite core samples (outer layer 10.2 g–13.8 g; inner layer 32.7 g–55.8 g) with a modified Bligh and Dyer protocol (43), using a 1:2:0.8 ratio of dichloromethane/methanol/phosphate buffer. Lipids were separated using silica gel chromatography. The polar fraction was subjected to methanolysis under mildly alkaline conditions to convert all phospholipids to fatty acid methyl esters (FAMEs). These FAMEs were purified using secondary silica gel chromatography and identified on an Agilent 7890B gas chromatograph equipped with a DB5-MS column (30 m, 0.25 mm, 0.25 µm film thickness) coupled with an Agilent 5977B inert mass selective detector (quadruple). The splitless injection port temperature was 300°C with a column flow of 1.4 mL/min. The temperature program was as follows: oven hold at 50°C for 1 min, ramp 20°C/min to 120°C, ramp 4°C/min to 160°C, ramp 8°C/min to 300°C, then hold 5 min at 300°C. Peaks were identified using retention times and molecular weights in comparison to the National Institute of Standards and Technology MS database and bacterial reference standards (Bacterial Acid Methyl Ester mix and Supelco 37 Component FAME Mix; Sigma-Aldrich). The PLFA analysis of Year 1 borehole module bentonite samples were reported previously (13) but samples were re-analyzed in the present study using the Agilent 7890B gas chromatograph and Agilent 5977B detector as described above.

### DNA extraction

Genomic DNA from filter membranes and swabs was extracted using the PowerSoil DNA Isolation Kit (Qiagen, previously MO BIO Laboratories, CA, USA) as described previously (13). Total genomic DNA from 2 g inner layer, outer layer, or powdered bentonite was extracted using the PowerMax DNA Isolation Kit. Extracted DNA was eluted in 2 mL of 10 mM Tris as described previously (13), omitting the final nucleic acid precipitation step. Purified DNA from cultivable heterotrophic bacteria and SRB were extracted using the DNeasy Ultraclean Microbial Kit (Qiagen). Colonies from R2A agar plates were resuspended in 100 µL of sterile DNA-free water. Two milliliters of liquid from MPN tubes was centrifuged at 10,000 × *g* for 5 min and the pellet was resuspended in 100 µL sterile DNA-free water. Genomic DNA was extracted following the manufacturer’s instruction using a bead beater (FastPrep-24 Instrument MP Biomedicals, USA) at 5.5 m/s for 45 s instead of vortex. Replicate DNA extracts (R2A plates or MPN tubes) were pooled before 16S rRNA gene PCR amplification. The Qubit dsDNA High Sensitivity Assay Kit (Invitrogen, CA, USA) with fluorescence measured on a Qubit 4.0 fluorometer (Life Technologies, CA, USA) was used to measure DNA concentrations.

### Quantitative 16S rRNA gene PCR

Bacterial 16S rRNA gene copies in bentonite DNA extracts were determined using 341F (CCTACGGGAGGCAGCAG) and 518R (ATTACCGCGGCTGCTGG) (44). The 15 µL reaction mixture contained 1× SsoAdvanced Universal SYBR green Supermix (Bio-Rad), 0.3 µM 341F primer, 0.3 µM 518R primer, 7.5 µg bovine serum albumin (BSA, UV-treated), and 4 µL DNA extract. The triplicate qPCR was performed on a CFX96 Real-Time PCR detection system (Bio-Rad) using 98°C for 3 min initial denaturation, 40 cycles of 98°C for 15 s, and 55°C for 30 s. A 10-fold serial dilution of the 16S rRNA gene of *Thermus thermophilus* was used as standard curve as described previously (8). Amplification efficiencies of 99.4% and 103.6%, and coefficients of determination (*R* ^2^) of 0.993 and 0.994 were obtained. The average of five to eight replicates of sample material was reported for each bentonite sample.

### Eukaryotic 18S rRNA gene PCR

The V4–V5 region of the eukaryotic 18S rRNA genes was amplified from bentonite sample DNA extracts using primers 574*F (CGGTAAYTCCAGCTCYV) and 1132R (CCGTCAATTHCTTYAART) (45). All 25 µL PCR amplifications contained 1× ThermoPol Buffer, 0.2 µM 574*F, 0.2 µM 1132R primer, 200 µM deoxyribonucleotide triphosphates (dNTPs), 15 µg BSA, 0.625 units Hotstart Taq DNA polymerase (New England Biolabs, MA, USA), and 2 µL DNA extract. The PCR was performed on a T100 PCR thermal cycler system (Bio-Rad) using 95°C for 3 min, followed by 45 cycles of 95°C for 30 s, 50°C for 30 s, 68°C for 1 min, and 68°C for 7 min.

### High-throughput amplicon sequencing

Primers 515F-Y (46) and 926R (47) were used to amplify the V4–V5 region of 16S rRNA genes. In addition to the 16S rRNA gene-specific regions, forward and reverse primer contained a unique 6 bp index and Illumina sequencing specific sequences (48). The 25 µL PCR contained 1× ThermoPol Buffer, 0.2 µM of each primer, 200 µM dNTPs, 15 µg BSA, 0.625 units Hotstart Taq DNA polymerase (New England Biolabs, MA, USA), and 2 µL DNA extract (randomized). Samples were amplified in triplicate using 95°C for 3 min, 45 cycles of 95°C for 30 s, 50°C for 30 s, 68°C for 1 min, and a final extension of 68°C for 7 min. DNA extracts from cultures were amplified as described above with 35 cycles. Triplicate reactions were pooled, quantified in an agarose gel, and equal nanogram quantities of amplicons were pooled to a maximum volume of 20 µL. A volume of 5 µL was pooled for each control (no template, kit, swab and Sterivex controls), even though no amplicons were detected. Pooled 16S rRNA gene amplicons were excised from an agarose gel, purified using the Wizard SV Gel and PCR Clean-Up System (Promega, WI, USA), and quantified using the Qubit dsDNA High Sensitivity Assay Kit (Invitrogen, CA, USA). The 8.5 pM library was sequenced on a MiSeq (Illumina, CA, USA) using a 2 × 250 cycle MiSeq Reagent Kit v2 (Illumina, NB, Canada) with 15% PhiX Control v3 (Illumina Canada).

### Bioinformatic and statistical analysis

Sequencing reads were demultiplexed according to index sequence using MiSeq Reporter software (version 2.5.0.5, Illumina). Reads were processed and analyzed using Quantitative Insights Into Microbial Ecology 2 [version 2021.2, (49)] managed by Automation, eXtension, and Integration Of Microbial Ecology [AXIOME3 (50)]. Within the AXIOME3 pipeline, DADA2 [release 1.10.0 (51)] removed low quality reads and chimeras, trimmed primer sequences, truncated forward and reverse reads to 250 bases, denoised, and dereplicated the data set. The AXIOME3 pipeline assigned taxonomy to ASVs using a naive Bayes classifier (classify-sklearn) trained on SILVA version 138 (52). Ordinations and triplots were generated using AXIOME3 using ASV tables rarefied to 2,000 reads per sample.

Negative controls (i.e., DNA extractions without sample added or clean/unused swabs or Sterivex membranes; PCR no template controls) were used to identify contaminant sequences using the decontam (53) prevalence method with a 0.5 score statistic threshold value. The decontam analysis was performed on data sets grouped by DNA extraction kit usage and batch (PowerMax, PowerSoil DNA Isolation kit) to aid identification of kit-related contaminants. A total of 59 ASVs were identified as contaminants by decontam (Fig. S1) and were removed from the ASV table.

Differential abundance analysis between Year 1, Year 5, or uncompacted bentonite was performed using analysis of composition of microbiomes (54).

## Data Availability

Sequences are available in the European Nucleotide Archive under accession number PRJEB48629, and additional metadata are provided in Table S2.

## SUPPLEMENTAL MATERIAL

Supplemental Material msphere.00048-23-s0001.docx Fig. S1 to S6.

Table S1 msphere.00048-23-s0002.xlsx ASV table for bentonite and borehole module samples.

Table S2 msphere.00048-23-s0003.xlsx ENA sample accessions and metadata.

ASM does not own the copyrights to Supplemental Material that may be linked to, or accessed through, an article. The authors have granted ASM a non-exclusive, world-wide license to publish the Supplemental Material files. Please contact the corresponding author directly for reuse.
