# Supplementary material for: Stable microbial community in compacted bentonite after 5 years of exposure to natural granitic groundwater
Source: mSphere. 2023 Sep 29;8(5):e00048-23. doi: 10.1128/msphere.00048-23 (PMC10597416; doi:10.1128/msphere.00048-23)
Supplement: Supplemental Material — Fig. S1 to S6. [file msphere.00048-23-s0001.docx]

Supplemental information

Stable microbial community in compacted bentonite after five years of exposure to natural granitic groundwater

Katja Engel^1^, Sian E. Ford^2^, W. Jeffrey Binns^3^, Nikitas Diomidis^4^, Greg F. Slater^2^, and Josh D. Neufeld^1^*

^1^Department of Biology, University of Waterloo, Waterloo, Ontario, Canada

^2^School of Geography & Earth Sciences, McMaster University, Hamilton, Ontario, Canada

^3^Nuclear Waste Management Organization, Toronto, Ontario, Canada

^4^NAGRA, Wettingen, Switzerland

^*^Corresponding author: Department of Biology, University of Waterloo, 200 University Avenue West, Waterloo, Ontario, N2L 3G1, Canada. Tel. +1 519-888-4567; Fax +1 519-746-0614.

E-mail: [jneufeld@uwaterloo.ca](mailto:jneufeld@uwaterloo.ca)

Keywords: MX-80 bentonite clay, nuclear waste disposal, microbial characterization, 16S rRNA gene sequencing, cultivation, PLFA

Supplemental figures


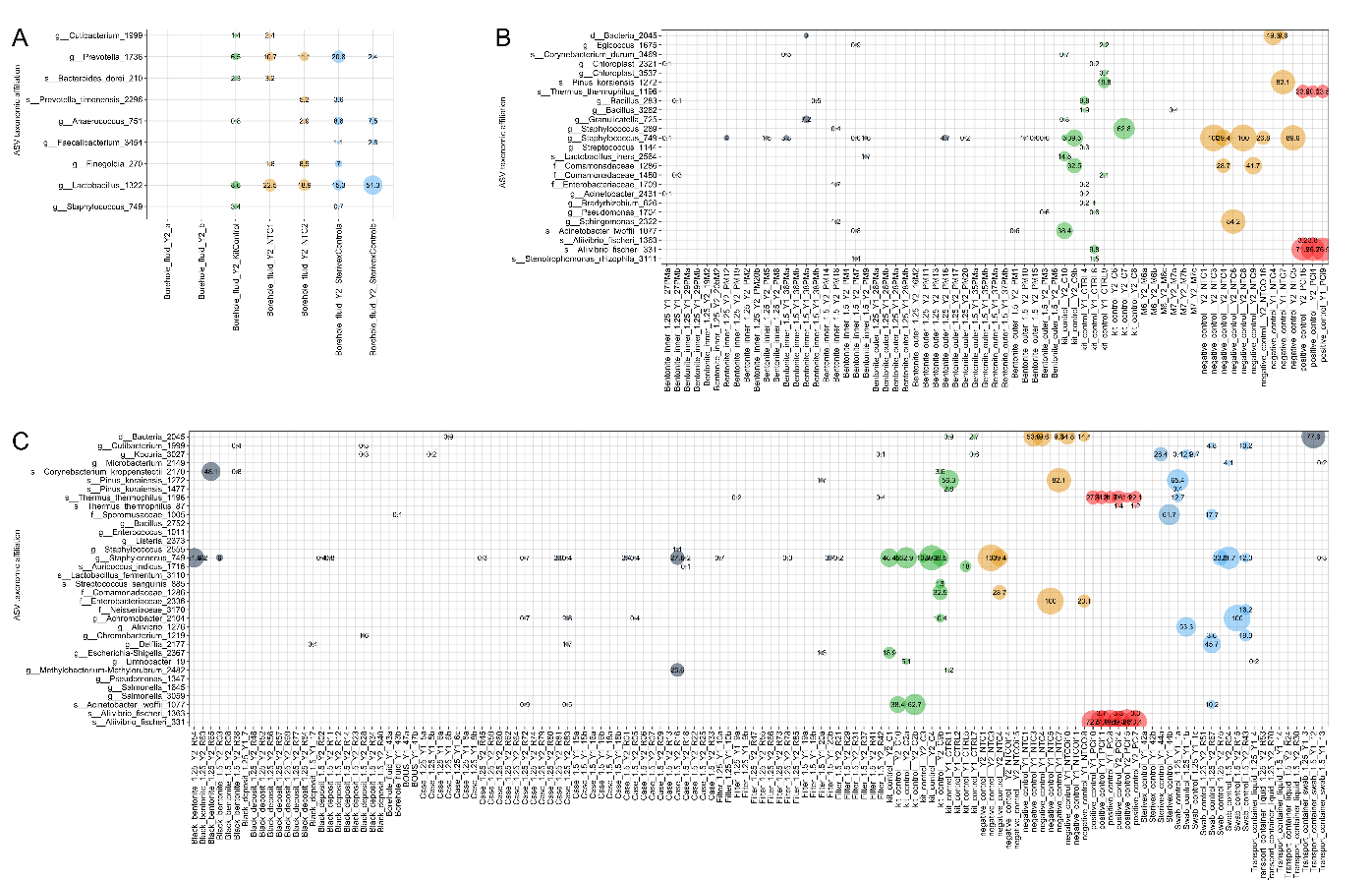


Figure S1. Bubble plot showing the contaminant ASVs identified by Decontam (threshold value of 0.5) and their relative abundance in samples extracted with following kits: PowerMax DNA Isolation batch #1 (A), PowerMax DNA Isolation batch #2 (B), and PowerSoil DNA Isolation (C). Only ASVs at or above 0.1% relative abundance are shown.


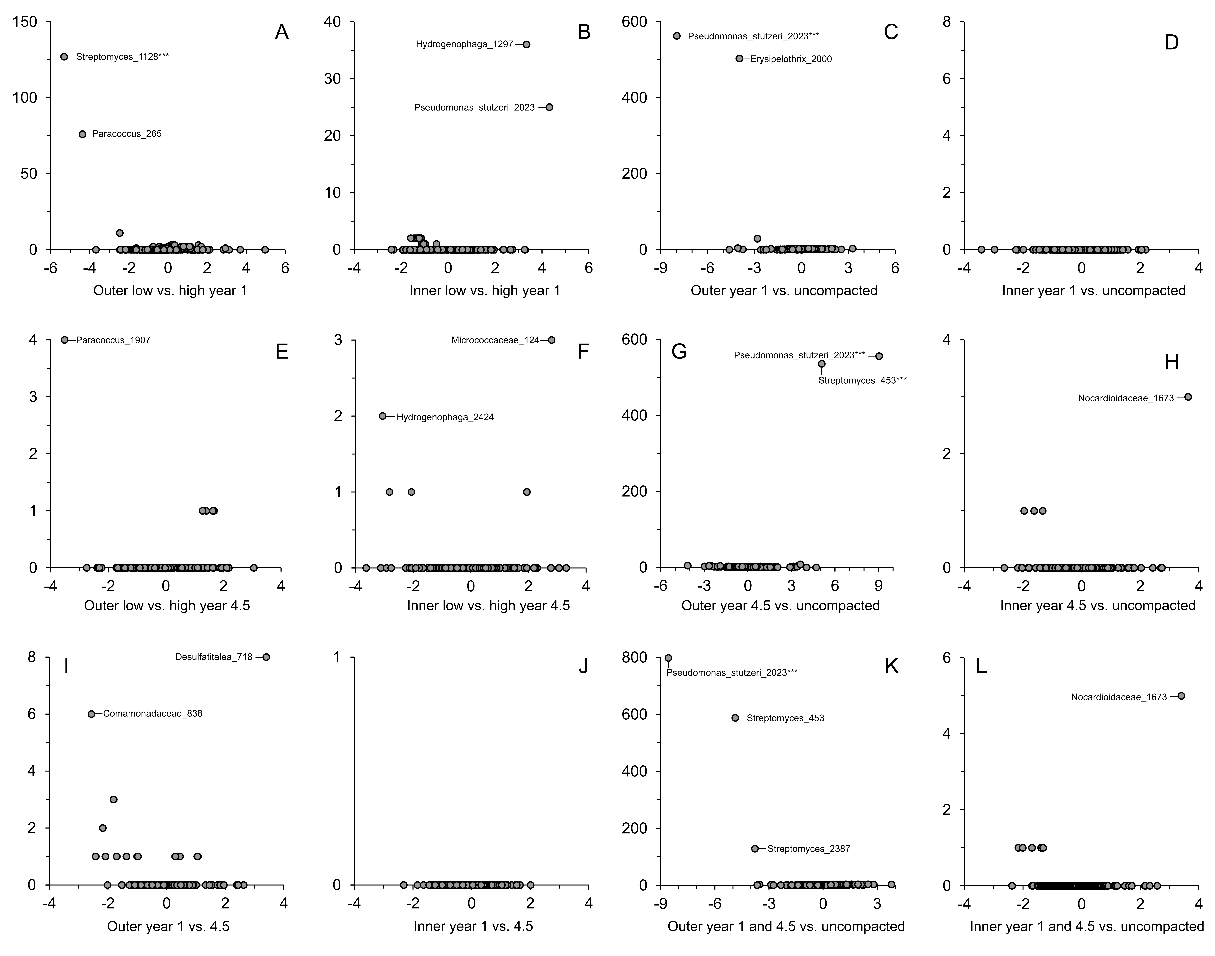


Figure S2. ANCOM differential abundance analysis of outer or inner layers of MaCoTe bentonite samples after 1 or 5 years of storage in borehole 13.001 and uncompacted bentonite used to prepare the modules. ASV #1128 (A), #2023 (C, G, K) and #453 (G) showed a statistically significant difference in abundance (*p* < 0.05, ***) between materials indicated in the x-axis label of the figure panel.


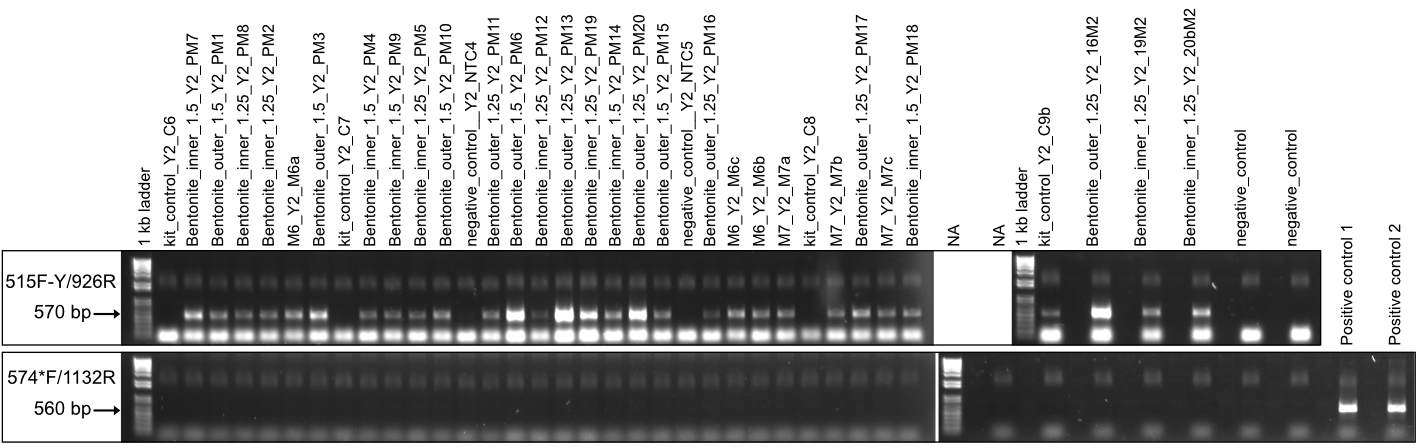


Figure S3. Agarose gel (1%) of the ~570 bp Illumina 515F-Y/926R 16S rRNA gene amplicons (top) and ~560 bp 574*F/1132R 18S rRNA amplicons (bottom) of bentonite DNA extracts from 1.25 or 1.5 g/cm^3^ dry density modules. DNA extraction controls (kit_control), PCR controls (negative_control) and 1kb DNA ladder (Invitrogen) are shown. Soil DNA extracts were used as positive control 1 and 2 for 18S rRNA gene PCR.


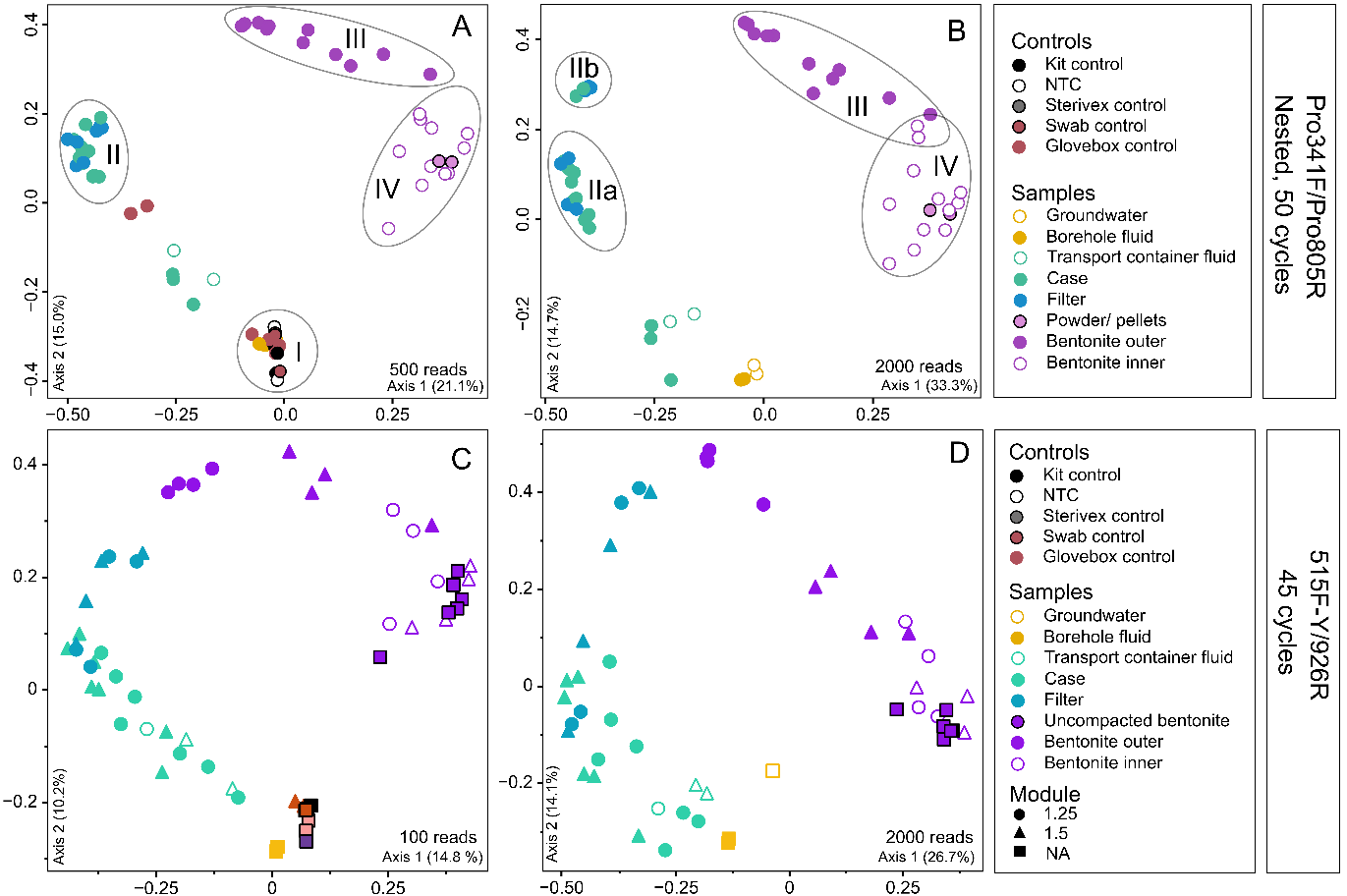


Figure S4. Grouping of year 1 borehole module samples in a PCoA ordination based on Bray-Curtis dissimilarities. Plot was generated by including (A, C) or excluding (B, D) controls. Samples were amplified using Pro341F/Pro518R (A, B) or 515F-Y/926r (C, D) primer pairs.


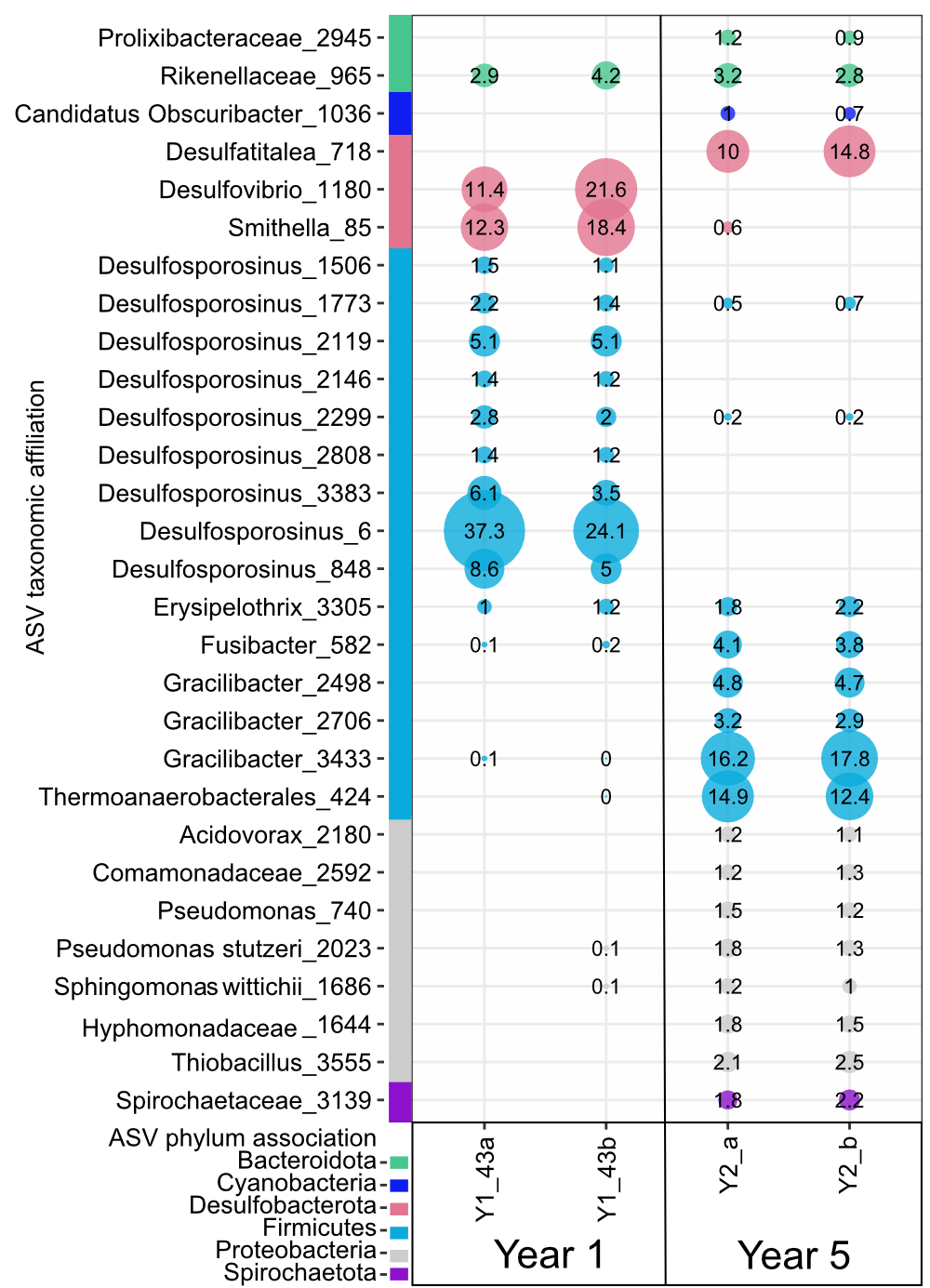


Figure S5. Bubble plot showing 16S rRNA gene profiles of borehole after 1 and 5 years of exposure. ASVs at or above 1% abundance in at least one sample are shown and lower abundances in other samples.


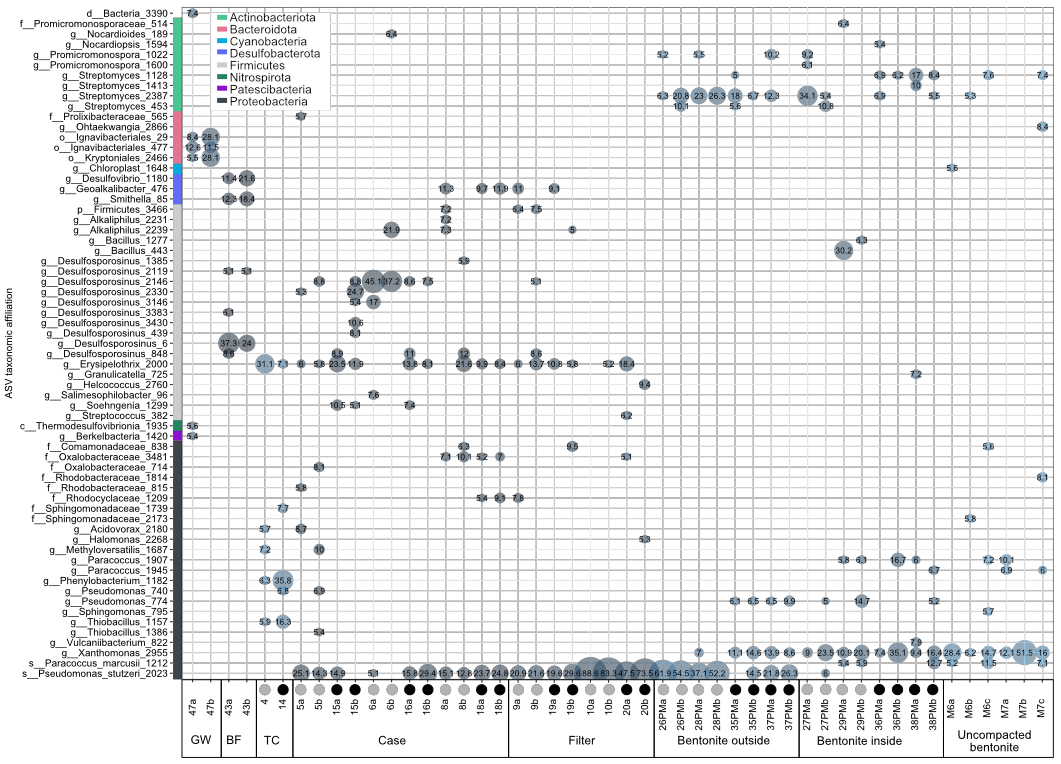
Figure S6. Bubble plot showing 16S rRNA gene profiles of borehole module and bentonite samples after 1 year of exposure in borehole 13.001. Only ASVs at or above 5% abundance are shown. Samples are sorted from the outside (natural ground water (GW), borehole fluid (BF), transport container (TC)) to the inside of the 1.25 (grey circles) and 1.50 (black circles) g/cm^3^ dry density borehole modules. The 16S rRNA gene profiles of uncompacted bentonite used to prepare the borehole modules are shown on the far right.

Supplemental table

Table S1. An ASV table showing abundances and representative sequence of ASVs in Year 5 borehole module samples. The ASV table was used to generate Figure 4.
